# The effect of sampling health facilities on estimates of effective coverage: a simulation study

**DOI:** 10.1186/s12942-022-00307-2

**Published:** 2022-12-17

**Authors:** Emily D. Carter, Abdoulaye Maiga, Mai Do, Glebelho Lazare Sika, Rosine Mosso, Abdul Dosso, Melinda K. Munos

**Affiliations:** 1 Institute for International Programs, Johns Hopkins Bloomberg School of Public Health, 615 North Wolfe Street, Baltimore, MD USA; 2 Department of Global Community Health and Behavioral Sciences, Tulane University School of Public Health and Tropical Medicine, Tulane, New Orleans, LA USA; 3 Ecole Nationale Supérieure de Statistique Et d’Economie Appliquée, Abidjan, Ivory Coast; 4 Johns Hopkins Center for Communication Programs, Abidjan, Ivory Coast

**Keywords:** Sampling, Census, Effective coverage, Quality-adjusted coverage, Simulation, GIS, Linking, Household survey, Facility assessment, Research methods

## Abstract

**Background:**

Most existing facility assessments collect data on a sample of health facilities. Sampling of health facilities may introduce bias into estimates of effective coverage generated by ecologically linking individuals to health providers based on geographic proximity or administrative catchment.

**Methods:**

We assessed the bias introduced to effective coverage estimates produced through two ecological linking approaches (administrative unit and Euclidean distance) applied to a sample of health facilities. Our analysis linked MICS household survey data on care-seeking for child illness and childbirth care with data on service quality collected from a census of health facilities in the Savanes region of Cote d’Ivoire. To assess the bias introduced by sampling, we drew 20 random samples of three different sample sizes from our census of health facilities. We calculated effective coverage of sick child and childbirth care using both ecological linking methods applied to each sampled facility data set. We compared the sampled effective coverage estimates to ecologically linked census-based estimates and estimates based on true source of care. We performed sensitivity analyses with simulated preferential care-seeking from higher-quality providers and randomly generated provider quality scores.

**Results:**

Sampling of health facilities did not significantly bias effective coverage compared to either the ecologically linked estimates derived from a census of facilities or true effective coverage estimates using the original data or simulated random quality sensitivity analysis. However, a few estimates based on sampling in a setting where individuals preferentially sought care from higher-quality providers fell outside of the estimate bounds of true effective coverage. Those cases predominantly occurred using smaller sample sizes and the Euclidean distance linking method. None of the sample-based estimates fell outside the bounds of the ecologically linked census-derived estimates.

**Conclusions:**

Our analyses suggest that current health facility sampling approaches do not significantly bias estimates of effective coverage produced through ecological linking. Choice of ecological linking methods is a greater source of bias from true effective coverage estimates, although facility sampling can exacerbate this bias in certain scenarios. Careful selection of ecological linking methods is essential to minimize the potential effect of both ecological linking and sampling error.

## Background

With the United Nations’ adoption of the Sustainable Development Goals (SDGs), including universal access to quality essential healthcare services, there has been an increased demand for effective coverage measures of health interventions that reflect both the use and quality of services within the target population. Effective coverage measures assess the proportion of individuals in need of intervention who receive it with sufficient quality to achieve the intervention's health benefit [1].

Linking household and service provider data is a valuable method for generating effective coverage measures. Existing household surveys, such as the Demographic and Health Survey (DHS, dhsprogram.com/Methodology/Survey-Types/DHS.cfm) and Multiple Indicator Cluster Survey (MICS, mics.unicef.org), capture a representative sample of the target population and provide data on care-seeking behavior. Health facility assessments, such as the Services Provision Assessment (SPA, dhsprogram.com/methodology/Survey-Types/SPA.cfm) and Service Availability and Readiness Assessment (SARA, https://www.who.int/data/data-collection-tools/service-availability-and-readiness-assessment-(sara)), capture data on structural (readiness) and process (provision of care) quality available to the target population. Combining data from these two sources can generate population-based estimates of intervention coverage adjusted for the likely quality of services received.

While numerous analyses have attempted to combine data from these sources, the methods for linking these data, constructing indicators, and interpreting results are highly variable [2]. The most common and feasible approach is to ecologically link households with health care providers based on geographic proximity or administrative catchment [3]. If data on the specific provider utilized is available from both a household and facility survey, then exact-match linking can be used to assign an individual to their true source of care. In the absence of data on where an individual accessed care, ecological linking assumes the individual utilized one or more providers within a close geographic range or defined administrative catchment. The implications of the design of existing household (DHS and MICS) and health facility surveys (SPA and SARA) for linking analyses are unknown. Sampling of health facilities has the potential to impact on effective coverage estimates generated using different ecological linking approaches.

The objective of linking analyses is to accurately capture the service environment in which the target population accessed care. Methods for ecological linking of individuals to service providers, based on geographic proximity or other variables, intend to effectively reflect service quality in the absence of data on the specific health provider utilized. However, if only a sample of health facilities are included in the facility data set, this may reduce the accuracy of linked analyses and bias effective coverage estimates.

Previous analyses have considered using geospatial data to combine household surveys and facility assessment data to generate covariates for service use or health outcome analyses. Skiles and colleagues found using a sample of health facilities (compared to a census) resulted in misclassification of relative service environment more than half of the time. Facility sampling was a greater source of misclassification error than household cluster displacement (i.e., random recoding of household cluster locations), and more geographically restrictive linking methods (e.g., the maximum allowed distance between a household and facility was capped) were more susceptible to misclassification [4]. Other analyses have warned against linking household and facility data below the sample domain level [5]. The DHS discourages analyses using distance-based covariates, as these methods are more likely to introduce error due to both cluster displacement and facility sampling [6]. However, linking households to the closest provider is still a commonly utilized approach.

No studies have explicitly looked at the effect of sampling on effective coverage estimation. We ran a simulation study on a unique data set from Cote d'Ivoire to assess facility sampling's impact on effective coverage estimation using two standard linking methods. This dataset is unique that in addition to collecting the standard household and facility data typically used in ecological effective coverage linking analyses, it also identified the specific health outlets utilized by survey participants allowing for more precise exact-match linking as a comparison.

## Methods

For this analysis, we used a data set of temporally and geographically concurrent household and health provider data collected in 2016 in the Savanes region of Cote d'Ivoire and used in a previous assessment of effective coverage estimation methods [7]. We used data on care-seeking collected from a sample of households and data on service readiness and process quality collected from a census of health care providers. We generated estimates of effective coverage for both sick child care and labor and delivery care. We used exact-match linking based on the reported provider utilized as our gold-standard estimate of effective coverage. We employed two ecological linking methods (1) linking a subject to the closest provider within the specified provider category and (2) assigning a subject the average quality score of all providers with the source of care category within a defined administrative unit. We then repeated the ecological linking analysis using a sample of facilities (drawn from our facility census) to simulate the effective coverage estimates that would be produced using standard SPA sampling methods. Comparing the census-based estimates to the sampled estimates by ecological linking method allows us to isolate facility sampling's effect on linked effective coverage estimates.

### Household survey

Household data were collected via an independently implemented MICS. The MICS collects data on services related to reproductive, maternal, neonatal, and child health. In Cote d'Ivoire, the 2016 MICS was sampled to be representative at the regional level [8]. For labor and delivery, women with a live birth in the last two years were asked about where they gave birth, classified by sector and facility level. For sick child care, women with a child under the age of five years with diarrhea, fever, or suspected acute respiratory infection (ARI) in the two weeks preceding the survey were asked about whether care was sought for the sick child and the source of that care, classified by sector and provider type. Additional questions were added to the standard survey asking respondents to specify the provider or facility they utilized for birth and/or sick child care. This information allowed us to link each subject to the individual health facility, outlet, or community-based provider from which care was sought. The central coordinates of each household cluster, as provided by the MICS, was used to approximate the location of sampled households.

### Healthcare provider assessment

In parallel with the MICS, we conducted a health provider assessment in the Savanes region. The assessment included all public, private, NGO, and religious first-level and referral facility-based providers, pharmacies, and pharmacy depots in the region. All CHWs serving the 44 sampled MICS household clusters in the region were also included in the assessment. We adapted the facility assessment tool from the SPA. The assessment collected data on infrastructure and commodities through a facility inventory, asked health workers about training and supervision, and assessed provision of care through direct observation of sick child consultations and health worker report of the interventions delivered during the last childbirth attended in the preceding 12 months. We used these data to generate estimates of facility inputs (structural quality) and quality (process quality) to provide labor and delivery and sick child care. We defined structural quality as the infrastructure, equipment, commodities, and human resources necessary to manage a given health issue. We consider process quality to be the correct assessment and management of patient needs by the health provider, gauged through observation of provision of care. The location of each facility, or CHW’s work primary site, was captured at the time of the survey.

We received ethical approval for the health facility assessment from the Johns Hopkins School of Public Health institutional review board (#00006896) and the Cote d’Ivoire Comité National d’Ethique de la Recherche (#025/MSLS/CNER-kp).

### Effective coverage estimation

We estimated the effective coverage of (1) sick child care and (2) labor and delivery (L&D) care. We estimated effective coverage by assigning each individual from the household survey the structural and process quality score of their linked source of care based on data from the health provider assessment. Applying the effective coverage cascades proposed by the Countdown to 2030 [9], a global collaboration for tracking progress towards the SDGs for women, children, and adolescents (countdown2030.org), we calculated input-adjusted coverage (care-seeking adjusted for facility structural quality) and quality-adjusted coverage (care-seeking adjusted for facility process quality). We implemented the linking analyses using the same methods outlined in the original effective coverage analysis by Munos and colleagues, including definitions of provider structural and process quality [7]. We used three linking methods in this analysis:

1. Exact-match linking: Each woman or sick child was linked to the specific provider they reported utilizing—collected via the additional questions in the MICS asking respondents to name their source of care.
2. Euclidean distance linking: Each woman or sick child was linked to the closest provider (as the crow flies) within the reported provider category (defined by managing authority and level of care).
3. Administrative unit linking: Each woman or sick child was assigned the aggregate score of all health providers with the administrative unit (district) and reported provider category, weighting for provider caseload.

We consider exact-match linking based on the specific, stated provider as the most precise method for linking a subject and source of care. We have used this measure as our "truth" comparator for gauging bias introduced by ecological linking and facility sampling. A previous analysis using this dataset compared both the Euclidean distance and administrative aggregate methods against the exact-match method. It found both approaches produced similar estimates of effective coverage in this setting when restricting by reported provider category and weighting for caseload [7]. We chose to examine these two ecological linking methods as they are commonly used approaches and performed well compared to the exact-match method when implemented using a provider census [7].

**Fig. 1:**
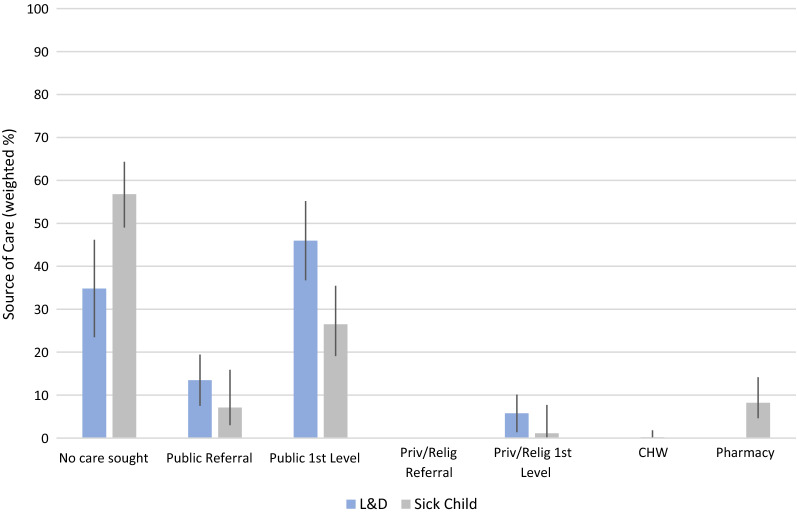
Source of sick child and delivery care reported in household survey

To reflect the type of data available from existing health facility assessments, we limited our analysis to use only health facility data. While we had data on both pharmacies and community-based health workers, standard SPA or SARA facility assessments do not include these sources of care. Exclusion of these sources had a minor effect on estimates of coverage in our prior analysis as both pharmacies and CHWs accounted for a small proportion of care-seeking for child illness (Fig. 1) and were not a source of care for labor and delivery [7].

In some cases, individuals could not be linked to a provider. Using the exact-match linking method, an individual would not be linked to their source of care if the respondent (1) could not recall the specific source of care, (2) the specified provider reported they did not provide the service the respondent stated they received, or (3) the provider assessment did not include the reported source of care. Despite using a census, a few providers that were not identified (primarily small private sector providers) for inclusion in the facility census or were outside of the study area. Using the ecological linking method, an individual would not be linked if there was no provider from the reported source of care category within the defined administrative area included in the assessment. In all cases, the subjects were assigned an average score for the management authority and level of facility they reported utilizing if they could not be linked.

While women could only give birth in a single facility, care for a sick child could be sought from multiple locations over the duration of the illness episode. While uncommon, children that sought care from more than one health provider were assigned the average input and quality scores of the providers they utilized. All GPS-related analyses, including measuring distance between points, were conducted in ArcGIS 10.8 (Esri, Redlands, CA, USA). All statistical analyses were conducted in Stata 16 (StataCorp, College Station, TX, USA).

### Sampling

We replicated the sampling methods used by the SPA as closely as possible. In line with standard SPA sampling methodology, we sampled among first-level facilities and maintained a census of referral facilities. Assuming the SPA was conservatively powered to estimate an indicator with ± 10% precision at the regional level, we estimated 65 facilities would need to be sampled. We also considered larger samples of 90 (precision ± 7.5%) and 130 (precision ± 5%) facilities. For each sample size, we drew 20 samples of facilities at random. We applied our administrative and closest provider linking analysis to generate effective coverage estimates for each sampled dataset.

In our dataset, there was limited variability in quality within facility categories. This limited the potential generalizability of our simulation results. Therefore, we ran two additional sensitivity analyses using simulated quality scores to assess the effect of sampling in settings with greater diversity in quality scores.

#### Quality simulation 1: random facility quality

In the first simulation, we maintained the data on facility type, facility location, and household care-seeking behavior. However, each facility was assigned a structural quality and process quality score completely at random (possible scores ranged from 0 to 1). Assigning a score at random was intended to increase the variability in provider scores within each provider category, increasing the opportunity that sampling could result in an individual being linked to facility with a substantially different score than their true source of care. The rest of the sampling and effective coverage estimation methods using both exact-match and ecological linking were implemented in the same manner as in the above section.

#### Quality simulation 2: preferential care-seeking from higher quality facilities

As in the previous simulation, we maintained the data on facility type, facility location, and household care-seeking behavior. However, each facility was assigned a structural quality and process quality score designed to simulate preferential care-seeking in favor of higher-quality facilities within a provider category. The rest of the sampling and effective coverage estimation methods were implemented in the same manner as in the above section. We assigned facilities that were utilized more frequently, based on the household survey data, a higher quality scores than those that were utilized less frequently or not at all. Facilities more than 10 km from a sampled MICS household cluster, and therefore unlikely to have been utilized by the MICS sample due to distance, had their score increased or decreased based on their performance relative to the median provider category score. The selected cut-offs and degree of score inflation/deflation were selected to (1) produce a plausible but sufficient spread in scores between high and low performing providers and (2) balance the number of providers whose scores were increased or decreased accounting for distance to prevent inherently biasing an ecological linking method.

Rules for altering facility quality scores from original Cote d'Ivoire data:

- If a facility was utilized by one or more (sick child) or two or more respondents (labor and delivery): increased facility quality by 15 percentage points
- If a facility was utilized by fewer than one (sick child) or two respondents (L&D) AND within 10 km of a sampled household cluster: decreased facility quality by 15 percentage points
- If a facility was utilized by fewer than one (sick child) or two respondents (L&D) AND more than 10 km from a sampled household cluster:
  o If facility score > the category median → increased facility quality by 15 percentage points
  p If facility score < category median → decreased facility quality by 15 percentage points

### Analysis

Our primary analysis compared estimates of input-adjusted and quality-adjusted effective coverage against (1) our best estimate of true effective coverage derived by exact-match linking and (2) the best estimate of effective coverage derived through ecological linking with a census of health providers. Comparing estimates from sampled datasets against the exact-match estimates quantifies the total bias introduced by both ecological linking and sampling providers. Comparing estimates from sampled datasets against ecological estimates using a census of providers isolates the bias introduced by facility sampling within an ecological linking method. Our analysis considers whether individual estimates derived through each approach differed significantly from either the exact-match estimate or census-derived ecological estimate. We also considered the distribution of the estimates to characterize the variability in estimates produced through each approach over 20 samples. Confidence intervals around the effective coverage estimates were derived from the sampling design and response rate of the household survey and treats the linked provider scores as an extension of the household survey dataset, ignoring error in the health facility assessment.

## Results

The provider assessment captured data on readiness and quality of care 144 public first-level facilities, 43 religious or private first-level facilities, and seven referral facilities (5 public and 2 private).

The MICS captured data on care-seeking for sick children and labor and delivery care from 44 household clusters in the Savanes region. This sample included 392 women who gave birth in the two years preceding the survey, among whom 65.2% (95% CI: 53.2–75.5%) delivered at a health facility. The sample also included 183 children under five who experienced fever, diarrhea, or symptoms of ARI in the two weeks preceding the survey. Among these children, care was sought for 43.2% (35.7–60.0%).

The most common source of care for sick children (Fig. 1) were public first-level facilities (27.0% of sick children), followed by pharmacies (8.2%). In our analysis, we considered pharmacies unskilled providers; we treated these children as receiving no skilled care if they did not seek care from another source. Just under half (46.0%) of women reported delivering at a first-level public facility (including maternity wards), followed by public referral facilities and private first-level facilities. No women reported seeking care from private referral facilities for delivery or sick child care. Using the exact-match linking method, 93.9% of sick children and 92.3% of delivering women could be assigned to their stated source of care.

### Real facility quality data

**Fig. 2:**
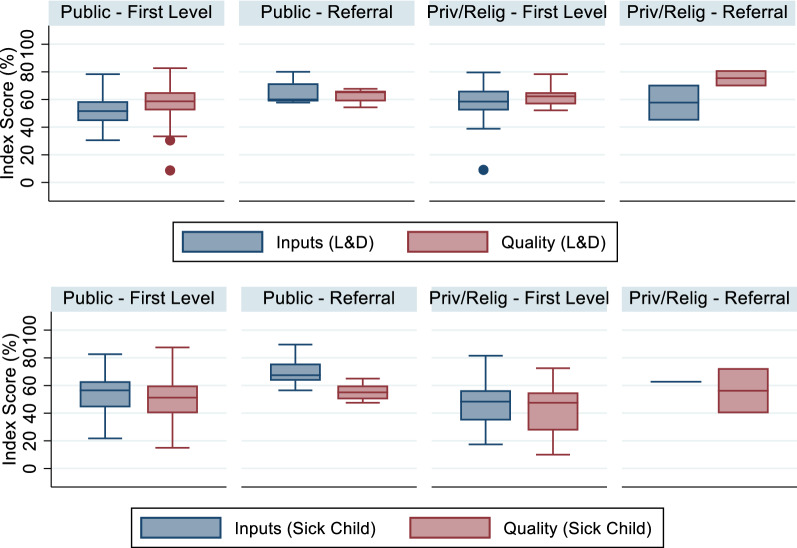
Distribution of facility scores, original Cote d'Ivoire data

Figure 2 shows the distribution of facility inputs and quality scores by provider type for sick child and delivery care. On average, referral facilities offer greater structural and process quality than first-level facilities. There is variation in individual first-level providers' quality, although the interquartile range (25^th^ to 75^th^ percentile) was typically less than 20 percentage points.

**Fig. 3:**
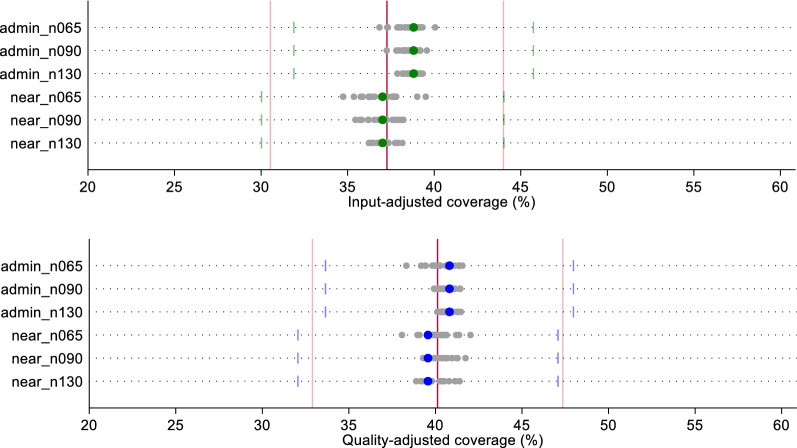
Estimates of input- and quality-adjusted coverage of labor and delivery care using original quality scores, by ecological linking method and facility sample size. The red line indicates the input- or quality-adjusted coverage based on exact-match linking. Light red lines indicate the 95% CI around the exact-match coverage estimate. Green (input-adjusted) and blue (quality-adjusted) dots indicate the coverage estimate derived from the ecological linking method applied to the census of providers. Light green (input-adjusted) and light blue (quality-adjusted) bars indicate the 95% CI around the census-derived ecologically linked effective coverage estimates. Gray dots indicate the effective coverage estimates produced using each of the 20 samples by sample size and ecological linking method

**Fig. 4:**
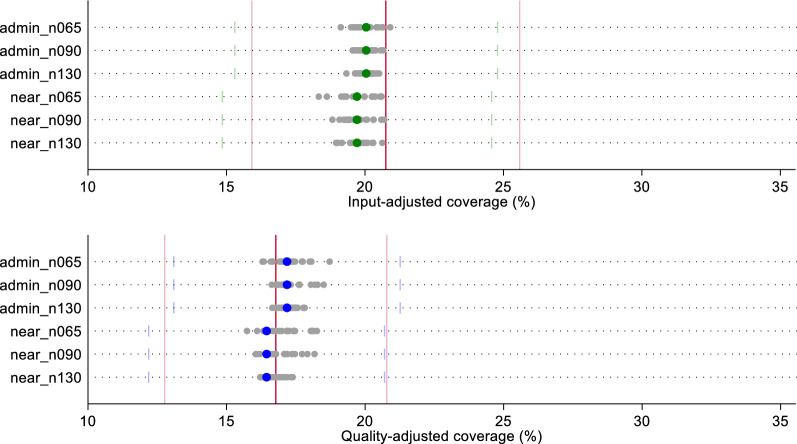
Estimates of input- and quality-adjusted coverage of sick child care using original quality scores, by ecological linking method and facility sample size

In Figs. 3 and 4, we compare the effective coverage estimate for labor and delivery care and sick child care, respectively, based on a census of providers using two linking methods (Euclidean distance or administrative unit average score) against the estimate generated using each random sample (n = 20) by linking method and sample size. Except for input-adjusted coverage for sick child care, the administrative linking method slightly overestimated exact-match coverage, and the Euclidean distance linking method slightly underestimated exact-match coverage. However, both ecological linking methods using a census of providers produced estimates of input- and quality-adjusted coverage that did not vary significantly from the exact-match linking estimates.

All sample-derived estimates fell within the confidence bounds of both the census-based ecological linked estimates and the exact-match estimates for labor and delivery care (Fig. 3). A greater spread in sample-derived estimates were observed for the smaller samples (n = 65); however, these estimates were still within the census estimates' bounds. We observed similar results with sick child care effective coverage estimates (Fig. 4). Despite greater spread in point estimates with smaller sample sizes, all sampled estimates fell within the confidence bounds of both the ecologically-linked census estimates and the exact-match linked estimates. Table 1 summarizes the effective coverage estimates using each of the linking and sampling methods.

**Table 1:** Summary of input- and quality-adjusted coverage estimates by linking method, facility sample size, and dataset

		Input-adjusted coverage		Quality-adjusted coverage	
		Admin link	Nearest link	Admin link	Nearest link
**Labor and delivery**					
* Original*					
Exact-Match	Mean (95% CI)	37.2 (30.5–43.9)		40.1 (32.9–47.3)	
Census	Mean (95% CI)	38.8 (31.9–45.7)	37.0 (30.0–44.0)	40.8 (33.6–48.0)	39.6 (32.0–47.1)
Sampled Datasets					
N = 65	Median [IQR]	38.4 [37.9–39.0]	37.1 [36.3–37.7]	40.4 [39.9–41.2]	40.2 [39.8–40.5]
N = 90	Median [IQR]	38.6 [38.3–38.9]	37.2 [36.2–37.8]	40.5 [40.2–41.0]	40.3 [39.6–40.9]
N = 130	Median [IQR]	38.8 [38.5–39.0]	37 [36.8–37.3]	40.6 [40.4–41.0]	39.8 [39.6–40.4]
* Random*					
Exact-Match	Mean (95% CI)	32.9 (25.5–40.2)		35.3 (27.4–43.2)	
Census	Mean (95% CI)	36.1 (29.3–42.9)	32.8 (24.8–40.8)	34.6 (28.2–41.0)	32.3 (24.1–40.5)
Sampled Datasets					
N = 65	Median [IQR]	36.0 [33.8–36.8]	35.3 [32.7–37.1]	34.8 [33.4–36.0]	32.6 [29.7–34.0]
N = 90	Median [IQR]	35.8 [34.6–36.8]	34.5 [33.0–35.4]	34.1 [32.8–35.1]	32.2 [30.2–33.5]
N = 130	Median [IQR]	35.9 [34.7–36.9]	34.5 [33.6–35.1]	34.7 [34.0–35.2]	32.6 [31.6–33.6]
* Preferential care-seeking*					
Exact-Match	Mean (95% CI)	45.1 (37.0–53.2)		48.0 (39.4–56.7)	
Census	Mean (95% CI)	43.5 (35.6–51.4)	43.2 (34.5–51.8)	45.2 (37.1–53.4)	45.7 (36.6–54.8)
Sampled Datasets					
N = 65	Median [IQR]	43 [42.1–43.8]	39.2 [38.2–40.8]	43.6 [42.8–45.8]	42.1 [41.1–43.4]
N = 90	Median [IQR]	43.1 [42.9–43.8]	40.8 [39.5–42.2]	44.6 [43.5–45.5]	43.2 [42.5–45.4]
N = 130	Median [IQR]	43.5 [43–44]	40.9 [39.9–42.4]	45 [44.5–45.5]	44.4 [42.4–45.6]
**Sick child**					
*Original*					
Exact-Match	Mean (95% CI)	20.8 (15.9–25.6)		16.8 (12.8–20.8)	
Census	Mean (95% CI)	20.0 (15.3–24.8)	19.7 (14.8–24.6)	17.2 (13.1–21.3)	16.4 (12.16–20.7)
Sampled Datasets					
N = 65	Median [IQR]	20.1 [19.7–20.5]	19.7 [19.3–20.3]	17.3 [16.9–17.5]	17.2 [16.6–17.6]
N = 90	Median [IQR]	19.9 [19.8–20.3]	19.7 [19.4–20.0]	17.2 [17.0–17.6]	16.7 [16.5–17.2]
N = 130	Median [IQR]	20.2 [19.9–20.3]	19.7 [19.5–20.0]	17.2 [17.0–17.4]	16.7 [16.5–17.1]
* Random*					
Exact-Match	Mean (95% CI)	19.8 (14.5–25.1)		19.2 (14.7–23.8)	
Census	Mean (95% CI)	19.9 (14.7–25.1)	19.4 (13.8–25.0)	18.8 (13.7–23.9)	18.4 (13.0–23.8)
Sampled Datasets					
N = 65	Median [IQR]	19.9 [19.3–20.7]	18.2 [17.3–19.5]	18.1 [17.6–19]	18 [17–18.4]
N = 90	Median [IQR]	19.6 [19.3–20.5]	18.7 [17.7–20.2]	18 [17.8–18.9]	17.8 [17.1–18.7]
N = 130	Median [IQR]	20.1 [19.5–20.3]	19 [18.6–19.4]	18.4 [17.9–19.1]	17.8 [17.1–18.3]
* Preferential care-seeking*					
Exact-Match	Mean (95% CI)	25.6 (19.6–31.5)		21.6 (16.5–26.6)	
Census	Mean (95% CI)	21.2 (16.0–26.4)	21.9 (16.1–27.7)	18.4 (13.9–23.0)	18.6 (13.4–23.9)
Sampled datasets					
N = 65	Median [IQR]	21.1 [20.6–21.6]	20.9 [20.3–21.7]	18.6 [17.9–19.4]	18.6 [17.7–20.1]
N = 90	Median [IQR]	21.1 [20.5–21.3]	20.8 [20.3–21.5]	18.8 [18.5–19.3]	18.5 [17.6–19.2]
N = 130	Median [IQR]	21.1 [20.8–21.4]	21.4 [20.4–22.1]	18.6 [17.9–19]	18.6 [18.2–18.9]

### Simulated random facility quality data

**Fig. 5:**
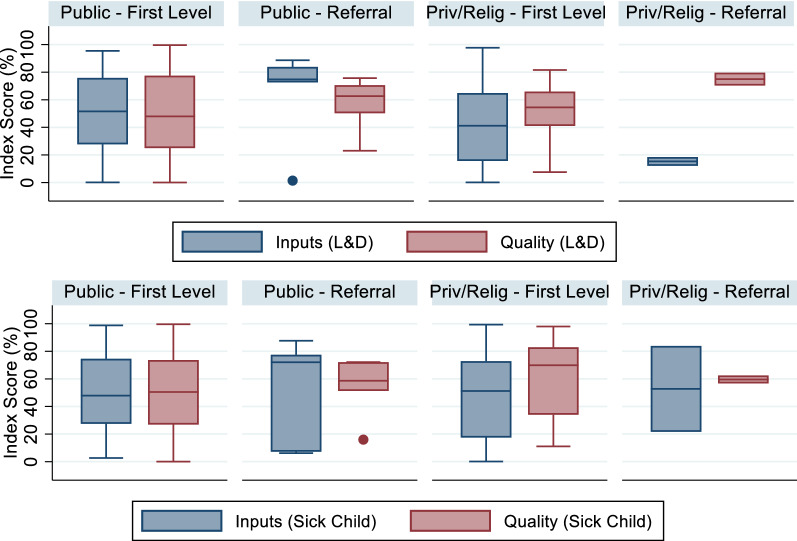
Distribution of facility scores, random index scores

Assigning each facility a structure and process-quality score at random, we estimated the effect of sampling facilities on effective coverage estimates in settings of high variability in provider quality. Figure 5 shows the distribution of facility input and quality scores for the facility census with random quality assignment. As expected based on the random assignment of facility scores, the median scores across for the two most numerous provider types (public and private first-level facilities) were approximately 50%, with an interquartile range of roughly 25% and 75%, respectively.

**Fig. 6:**
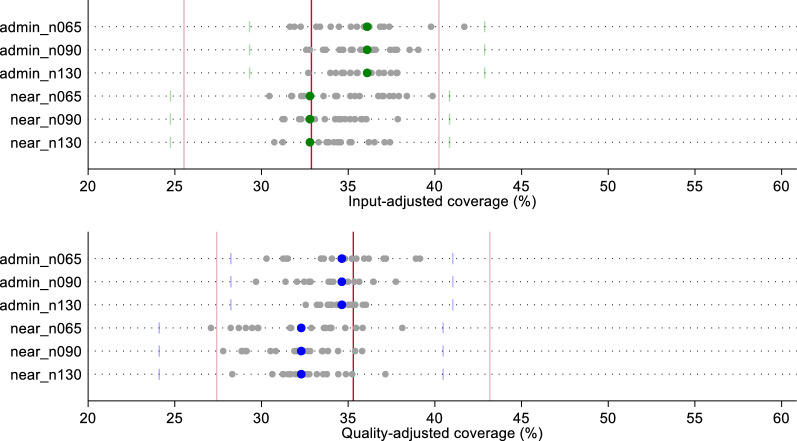
Estimates of input- and quality-adjusted coverage of labor and delivery care using random quality scores, by ecological linking method and facility sample size

**Fig. 7:**
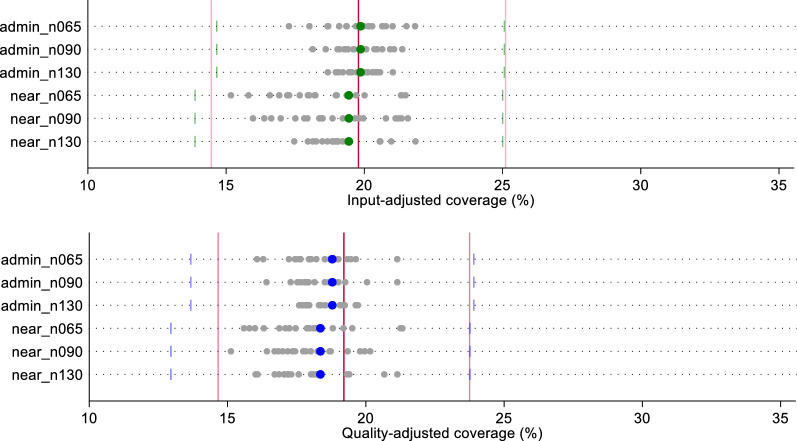
Estimates of input- and quality-adjusted coverage of sick child care using random quality scores, by ecological linking method and facility sample size

In Figs. 6 and 7, we compare the effective coverage estimate based on a census of providers using the two linking methods against the estimates generated using the samples of random index scores (n = 20) by linking method and sample size. All sample-derived estimates fell within the bounds of their respective census-derived ecologically linked value. For labor and delivery, one sampled estimate of input-adjusted coverage and quality-adjusted coverage each fell outside of the confidence bounds of the exact-match coverage estimate (1 outlier out of 60 simulations each, 1.7%). Both outlying estimates were a product of the smallest (n = 65) sample size. None of the sampled estimates of input- or quality-adjusted coverage of sick child care fell outside the exact-match estimate bounds. Input-adjusted coverage estimates derived from samples using Euclidean distance appear to over-estimate labor and delivery coverage and underestimate sick child care when comparing the median and IQR of sampled estimates against the census-derived estimates (Table 1). The spread in sampled estimates was generally greater for Euclidean distance-derived estimates compared to administrative average estimates. In total, only 0.4% (n = 2/480) of estimates fell outside of the bounds of the exact-match derived estimate, and none deviated significantly from the census-derived, ecologically linked estimates.

### Simulated preferential care-seeking from higher quality providers

**Fig. 8:**
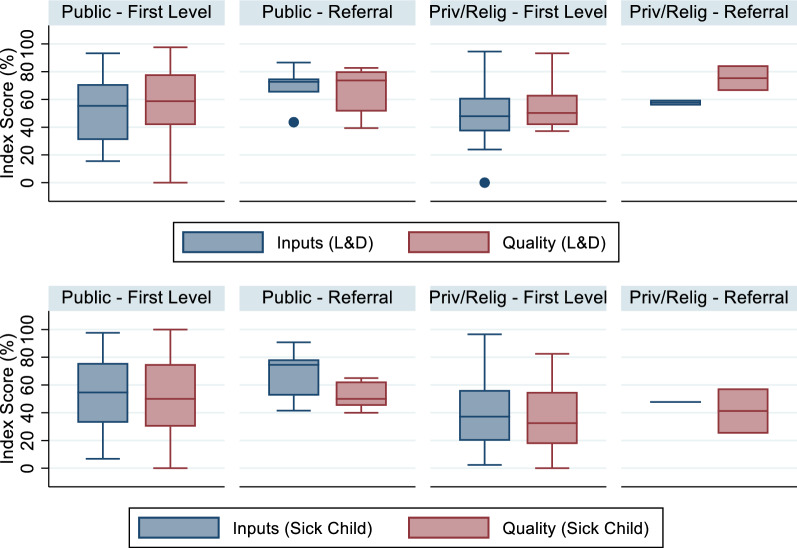
Distribution of facility scores, simulated preferential care-seeking from higher-quality providers

After simulating preferential care-seeking from higher quality providers using the original Cote d'Ivoire data set, we see a greater spread in structural and process quality scores within provider categories compared to the original dataset. We increased the score of the more heavily utilized facilities, while less used facilities have reduced scores. As in the original dataset, the simulated scores for referral facilities continue to be greater than first-level facilities. However, the interquartile range in first-level providers' scores increased to approximately 25 to 45 percentage points (Fig. 8). The median score for public first level facilities, the most utilized source of care, remained stable.

**Fig. 9:**
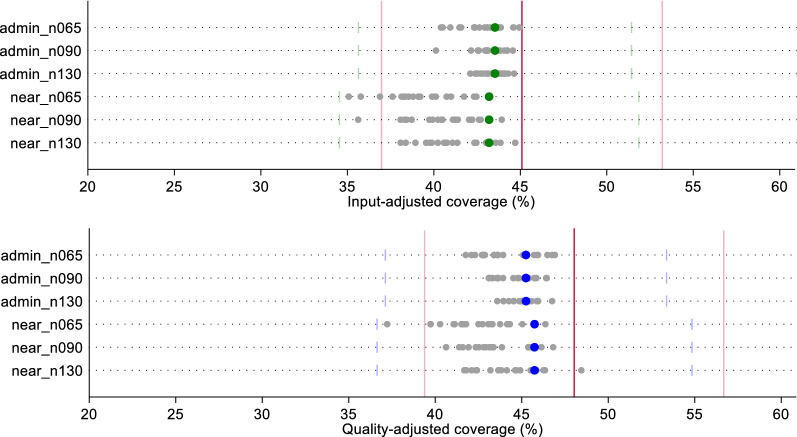
Estimates of input- and quality-adjusted coverage of labor and delivery care using simulated preferential care-seeking scores, by ecological linking method and facility sample size

**Fig. 10:**
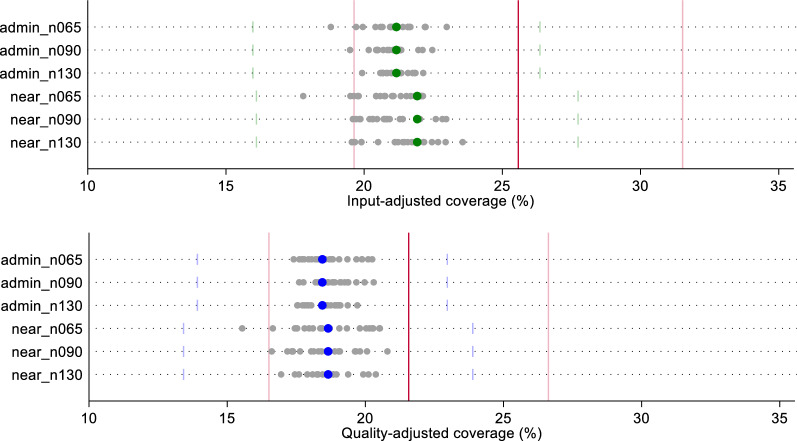
Estimates of input- and quality-adjusted coverage of sick child care using simulated preferential care-seeking scores, by ecological linking method and facility sample size

Ecological linking systematically underestimated the exact-match estimates using this preferential care-seeking simulated data set (Figs. 9, 10). The census and sample based ecological linking underestimated the exact-match point estimate for all linking methods, sample sizes, and outcomes. However, few estimates were statistically different from the exact-match coverage estimates. The smallest sample size resulted in 5.6% of estimates (9 outliers out of 160 simulations) falling below the exact-match estimate's lower bound (across health areas and outcomes). Both the moderate (n = 90) and large (n = 130) sample sizes resulted in outlying estimates in 1.2% of the iteration (n = 2/160 each). More outliers occurred using the Euclidean distance linking method (n = 11/240, 4.6%) versus the administrative linking (n = 2/240, 0.8%). The two administrative linking sampled estimates that fell outside the exact-match estimate bounds were estimates of input-adjusted sick child care estimated using a sample size of 65 and 90. Census estimates generated using the administrative linking approach were similarly biased against the exact-match estimate, compared to the Euclidian distance method. However, the sampled estimates derived using the Euclidian linking approach were more variable as demonstrated by the number outliers generated using the approach and the wider IQR of sampled estimates comparing Euclidean versus administrative linking methods by sample size and outcome (Table 1).

While 2.7% (n = 13/480) of our sampled estimates fell outside of the exact-match bounds, none of the sampled estimates fell outside of the bounds of the census-derived estimates generated through ecological linking. As observed with the other data sets, there was a greater spread in sample-derived estimates generated using smaller facility sample sizes.

## Discussion

Our analysis examined the effects of health facility sampling on effective coverage estimation. Sampling facilities from our health facility census data from the Savanes region of Cote d'Ivoire, we observed no statistically significant differences in input- or quality-adjusted coverage compared to either the ecologically linked estimates derived from a census of facilities or exact-match estimates. Similarly, there was little evidence of sampling altering effective coverage estimates when applied to our simulated data set with randomly assigned facility input and quality scores.

There was some evidence supporting underestimation of effective coverage when sampling health facilities in a setting where individuals preferentially sought care from higher-quality providers. In this simulation, 13 out of 480 estimates (2.7%) derived from samples fell outside of the confidence bounds of the exact-match input- or quality-adjusted coverage estimates. Both smaller sample size (9 of the 13 outliers were n = 65) and linking by Euclidean distance (11 out of 13 outliers) were associated with a greater occurrence of the estimate being an outlier. However, none of the sampled estimates fell outside of the bounds of the census-derived ecologically linked estimates. In this simulation, both ecological linking methods consistently underestimated the exact-match estimate. These findings suggest that the ecological linking method, rather than sampling, is the driver of the deviation from the "true" effective coverage estimate. However, the combination of sampling and ecological linking results in cases of effective coverage estimates that deviate significantly from the true (exact-match) effective coverage estimates and smaller sample sizes increased the potential for inaccurate estimates.

The administrative aggregate method was less susceptible to bias introduced through facility sampling. The current SPA sampling approach samples facilities at random within managing authority and level strata. This sampling introduces sampling error, as the sample may miss or include high or low performing facilities purely by chance. When using the administrative unit aggregate method, these chance omissions balance out when scores are averaged across the larger administrative unit. This results in less variability in the quality scores used in the ecological linking approach. An analysis by Skiles and colleagues similarly found that linking individuals to providers based on absolute distance misclassified relative service environment more frequently than using administrative unit linking in Rwanda [4].

In this setting, among those who sought care from a skilled provider, 50% of sick children and 54% of delivering women sought care from the closest provider (by Euclidean distance) within the level of care they reported utilizing [7]. About half of those who sought care therefore either bypassed a closer provider (within the same level of care), or Euclidean distance did not accurately reflect accessibility. Thus, the Euclidean distance ecological linking method misclassifies the source of care for approximately half of those who sought care from a skilled provider when a census of providers is used. In a setting where quality is relatively consistent within a level of care, incorrect linkages have little effect on quality-adjusted coverage estimates. However, where the quality of care is more variable, as introduced in our sensitivity analyses, linking an individual to the closest provider underestimates true coverage in settings where individuals bypassed lower quality facilities for a higher-performing provider. Sampling from the census of providers introduces additional error, as a smaller proportion of individuals are linked to their true source of care. Nevertheless, with a sufficiently large sample size random sampling should result in an approximately equivalent ratio of high and low performing providers drawn in each sample compared to a census of providers, minimizing the impact of incorrect linkages at a population level. However, the sample size can become insufficiently small to achieve balance when stratified by fine geographic units or other factors.

In the absence of weighting by provider volume, the existing sampling approach represents the landscape of providers, not the care received from a population perspective. Data on provider volume, which is not currently routinely collected with sufficient detail through facility assessments, can give a better picture of individual facility utilization by the population compared to household survey data alone which only shows the proportion of individuals utilizing broad provider categories. Sampling becomes an issue when, within a provider category used in the facility sampling and linking analysis, areas of high population density have better quality facilities that see a higher volume of patients. Suppose more remote, poorer quality providers are sampled in these situations rather than a better-performing urban facility. In that case, a greater proportion of the household sample will be incorrectly linked to lower-quality providers. In this fashion, sampling in combination with Euclidean distance linking together could contribute to underestimating effective coverage.

In the administrative unit linking approach, we weighted each sampled facility’s contribution to the overall aggregate category score by their relative facility caseload. We did not explicitly evaluate the contribution of weighting on the performance of the administrative linking method. However, our previous analysis using census data showed that weighting improved the performance of the method when compared against the exact-match estimates [7]. It could be expected that weighting higher volume facilities could at least partially adjust the facility sample to better reflect population-level use. Alternative methods for linking individuals to facilities exist, however, we limited this analysis to the two best performing linking methods employed in our previous analysis of this dataset [7]. Notably, we did not examine the use of shortest travel distance, which our previous analysis found resulted in linking fewer individuals to their true source of care compared to using Euclidean distance in this dataset.

Our analysis is limited by the lack of diversity in care-seeking behavior and within category provider quality in the study area. However, the high proportion of care-seeking from government facilities is similar to care-seeking behavior in much of sub-Saharan Africa [10]. Our sensitivity analyses using randomly assigned quality scores and simulated preferential care-seeking reflect the performance of these methods in settings with greater variability in provider quality and improve the generalizability of our findings. However, the findings may not hold in areas with a greater density of providers and diversity in care-seeking behaviors, supporting the need for additional research. Additionally, this analysis only considered delivery and sick child care. These points of care align with other maternal, neonatal, and child services, but may not be generalizable to other service areas such as HIV testing and treatment or management of chronic diseases. Our original estimates of provider quality have not been validated; however, they align with the types of indices commonly used in effective coverage measures.

The variance around the exact-match and census-based ecological effective coverage estimates were derived from the household survey. We assumed no sampling error in our measurement of facility quality as we collected data from a census of facilities. Inclusion of error from the facility data set would increase the variance of our effective coverage estimates and further reduce the cases where sampled estimates fell outside of the reference bounds. We only assessed the performance of these methods producing effective coverage estimates at the level of the survey sample domain, in this case, the Savanes region. While estimates should not be generated below the sample domain level, estimates of effective coverage are often incorrectly generated for smaller administrative units and the performance of these methods may not hold at those levels. Additionally, the smallest sample size we evaluated may be generous compared to the domain level sample sizes employed within some health facility assessments. Finally, our analysis used the central point of cluster locations in the Euclidean distance analysis. The DHS displaces cluster locations and this process could introduce additional bias into effective coverage estimates produced using DHS household survey data [4]. Our analysis did not explore the effect of cluster displacement in conjunction with health facility sampling on bias in effective coverage estimation.

## Conclusions

Our analyses suggest that current health facility sampling approaches do not significantly bias estimates of effective coverage produced through ecological linking, provided preferential care-seeking within a provider category is not common and a sufficiently large sample is drawn. The choice of ecological linking methods is a greater source of bias from true effective coverage estimates. However, facility sampling can exacerbate this bias in certain scenarios. Careful selection of ecological linking methods, such as restriction by provider type and weighting by provider volume, is essential for minimizing the potential effect of sampling error. In our analysis, administrative linking was less susceptible to chance deviation from the truth than the Euclidean distance method. In most scenarios, administrative linking did not bias estimates compared to exact-match linking supporting the validity of this approach which is often more feasible to implement than the more data and methods intensive exact-match linking. Further research is needed in other settings, including more urban populations and areas with more diverse provider landscapes, to better understand the performance of linking methods for effective coverage estimation. The development of standardized, best-practice guidance would serve to improve the validity and comparability of effective coverage estimates.

## Data Availability

The datasets analyzed during the current study are available from the corresponding author on reasonable request.

## Abbreviations

ARI: Acute respiratory infection
CHW: Community health worker
DHS: Demographic and Health Survey
IQR: Inter-quartile range
L&D: Labor and delivery
MICS: Multiple Indicator Cluster Survey
SARA: Service Availability and Readiness Assessment
SPA: Service Provision Assessment
SDGs: Sustainable development goals
